# Editorial: Mapping the exposome and investigating its role in human and environmental health

**DOI:** 10.3389/fmicb.2023.1353623

**Published:** 2024-01-05

**Authors:** Peng Gao, Feng Ju, Chao Jiang

**Affiliations:** ^1^Department of Environmental and Occupational Health, University of Pittsburgh, Pittsburgh, PA, United States; ^2^Department of Civil and Environmental Engineering, University of Pittsburgh, Pittsburgh, PA, United States; ^3^UPMC Hillman Cancer Center, Pittsburgh, PA, United States; ^4^Key Laboratory of Coastal Environment and Resources Research of Zhejiang Province, School of Engineering, Westlake University, Hangzhou, Zhejiang, China; ^5^Westlake Laboratory of Life Sciences and Biomedicine, Hangzhou, Zhejiang, China; ^6^Zhejiang Provincial Key Laboratory of Cancer Molecular Cell Biology, Life Sciences Institute, Zhejiang University, Hangzhou, Zhejiang, China; ^7^State Key Laboratory for Diagnosis and Treatment of Infectious Diseases, National Clinical Research Center for Infectious Diseases, First Affiliated Hospital, Zhejiang University School of Medicine, Hangzhou, Zhejiang, China

**Keywords:** exposome, environmental health, microbial communities, artificial intelligence, public health events

The concept of the exposome, encapsulating the totality of environmental exposures throughout an individual's life, presents a transformative perspective in understanding the intricate interplay between our surroundings and health. Recent studies in this field are pioneering diverse transdisciplinary approaches, shedding light on the multifaceted impacts of the exposome on both human and environmental health.

In the study conducted by Kim et al. which focused on the agricultural sector, the risks associated with ochratoxin A in the production of Angelicae Gigantis Radix were analyzed. This research highlighted the crucial role of monitoring and controlling environmental factors to ensure the safety of medicinal herbs. Their study underlines the necessity of stringent postharvest procedures to mitigate mycotoxin risks, emphasizing the broader implication of agricultural practices on health through the lens of the exposome.

In contrast, the investigation of early-life exposure to tobacco and mercury by Perez et al. explores the long-term health implications of toxic environmental factors. Their findings on the enduring impact of prenatal tobacco exposure on children's gut microbiota provide compelling evidence of how early-life environmental factors can shape health trajectories, reiterating the significance of minimizing harmful exposures during critical fetal developmental periods.

In expanding the horizons of exposome research, the study by Qiu et al. meticulously examines the effects of abamectin in agriculture, particularly its impact on soil microbial communities. Their research reveals that abamectin significantly alters the composition and interaction dynamics within these communities, leading to decreased biodiversity and stability. This disruption not only undermines soil health but also raises concerns about long-term health risks, notably the proliferation of antibiotic-resistant genes. These findings underscore the necessity for a critical reassessment of current agricultural practices, advocating for more sustainable and eco-friendly approaches in farming.

Yang et al. showcase advancements in environmental monitoring through the creation of the EMDS-7 dataset, utilizing cutting-edge artificial intelligence algorithms for the precise detection of environmental microorganisms. This methodological innovation leverages machine learning to identify and quantify microbial presence, significantly enhancing the accuracy and speed of environmental pollution assessments and their associated health impacts. The integration of AI not only exemplifies a significant advance in exposome research but also paves the way for cross-disciplinary interest, attracting scholars and practitioners from the fields of technology, environmental science, and public health.

Complementing these studies, Han et al. studied the impact of meteorological factors on scrub typhus in China, underscoring the need for a climate-informed approach to managing public health. Their work reveals how regional climatic conditions can influence disease prevalence, highlighting the necessity for targeted monitoring and control strategies in disease management.

These diverse studies collectively paint a comprehensive picture of the exposome, highlighting its vast implications across different sectors. From agriculture to public health, the exposome framework is crucial in addressing the complex relationships between environmental exposures and health outcomes of diverse organisms (Figure 1). As we delve deeper into this field, it becomes increasingly clear that an integrated approach, combining technological advancements with ecological and health considerations, is essential. This body of work not only enriches our understanding of the environment-health nexus but also lays the groundwork for innovative strategies to mitigate the adverse effects of environmental exposures.

**Figure 1:**
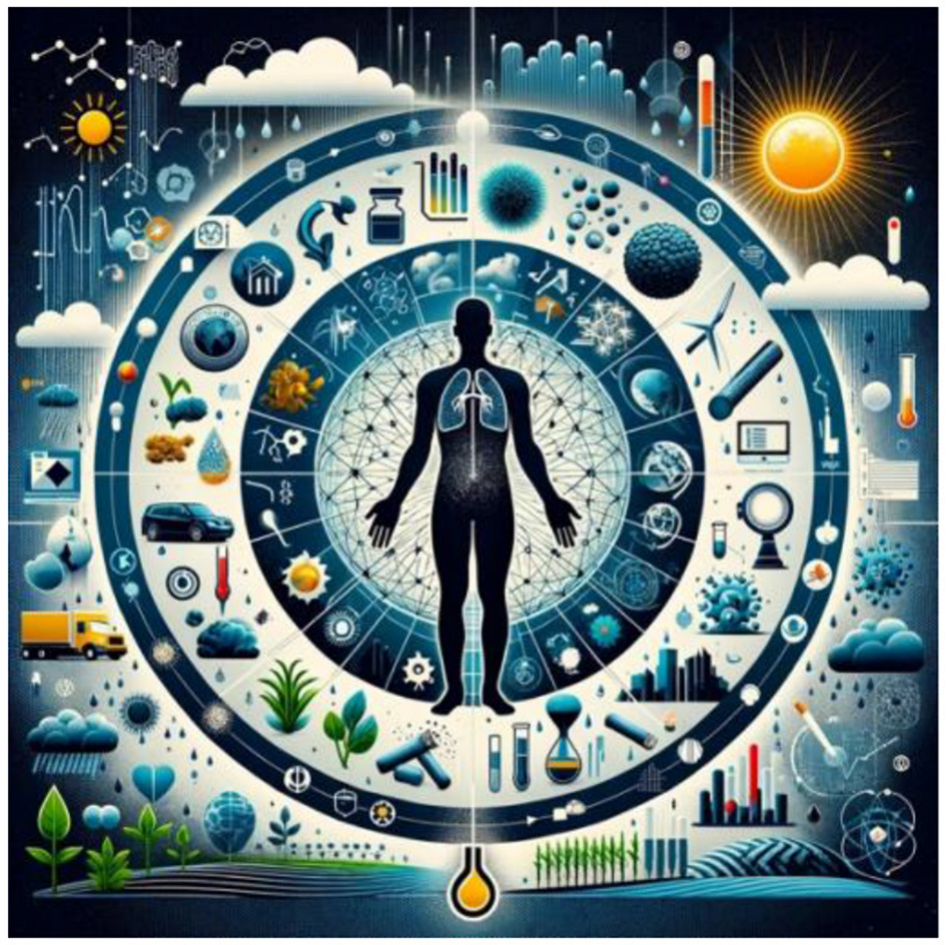
Interconnectedness of the Human Exposome: Environmental Influences on Health. The complex network of environmental influences surrounding a central human silhouette, represents the individual's health within the exposome. Elements surrounding the human such as agricultural, chemical, technological, and climatic factors, all interlinked to signify their cumulative impact on human well-being.

## Author contributions

PG: Conceptualization, Visualization, Writing—original draft, Writing—review & editing. FJ: Writing—review & editing. CJ: Writing—review & editing.

